# Quality Control in Targeted GC-MS for Amino Acid-OMICS

**DOI:** 10.3390/metabo13090986

**Published:** 2023-08-31

**Authors:** Dimitrios Tsikas, Bibiana Beckmann

**Affiliations:** Core Unit Proteomics, Institute of Toxicology, Hannover Medical School, 30623 Hannover, Germany

**Keywords:** amino acids, metabolites, OMICS, plasma, quality control, sample preparation

## Abstract

Gas chromatography-mass spectrometry (GC-MS) is suitable for the analysis of non-polar analytes. Free amino acids (AA) are polar, zwitterionic, non-volatile and thermally labile analytes. Chemical derivatization of AA is indispensable for their measurement by GC-MS. Specific conversion of AA to their unlabeled methyl esters (d_0_Me) using 2 M HCl in methanol (CH_3_OH) is a suitable derivatization procedure (60 min, 80 °C). Performance of this reaction in 2 M HCl in tetradeutero-methanol (CD_3_OD) generates deuterated methyl esters (d_3_Me) of AA, which can be used as internal standards in GC-MS. d_0_Me-AA and d_3_Me-AA require subsequent conversion to their pentafluoropropionyl (PFP) derivatives for GC-MS analysis using pentafluoropropionic anhydride (PFPA) in ethyl acetate (30 min, 65 °C). d_0_Me-AA-PFP and d_3_Me-AA-PFP derivatives of AA are readily extractable into water-immiscible, GC-compatible organic solvents such as toluene. d_0_Me-AA-PFP and d_3_Me-AA-PFP derivatives are stable in toluene extracts for several weeks, thus enabling high throughput quantitative measurement of biological AA by GC-MS using in situ prepared d_3_Me-AA as internal standards in OMICS format. Here, we describe the development of a novel OMICS-compatible QC system and demonstrate its utility for the quality control of quantitative analysis of 21 free AA and metabolites in human plasma samples by GC-MS as Me-PFP derivatives. The QC system involves cross-standardization of the concentrations of the AA in their aqueous solutions at four concentration levels and a quantitative control of AA at the same four concentration levels in pooled human plasma samples. The retention time (*t*_R_)-based isotope effects (IE) and the difference (δ(H/D) of the retention times of the d_0_Me-AA-PFP derivatives (*t*_R_(H)) and the d_3_Me-AA-PFP derivatives (*t*_R_(D)) were determined in study human plasma samples of a nutritional study (*n* = 353) and in co-processed QC human plasma samples (*n* = 64). In total, more than 400 plasma samples were measured in eight runs in seven working days performed by a single person. The proposed QC system provides information about the quantitative performance of the GC-MS analysis of AA in human plasma. IE, δ(H/D) and a massive drop of the peak area values of the d_3_Me-AA-PFP derivatives may be suitable as additional parameters of qualitative analysis in targeted GC-MS amino acid-OMICS.

## 1. Introduction

**Figure 1:**
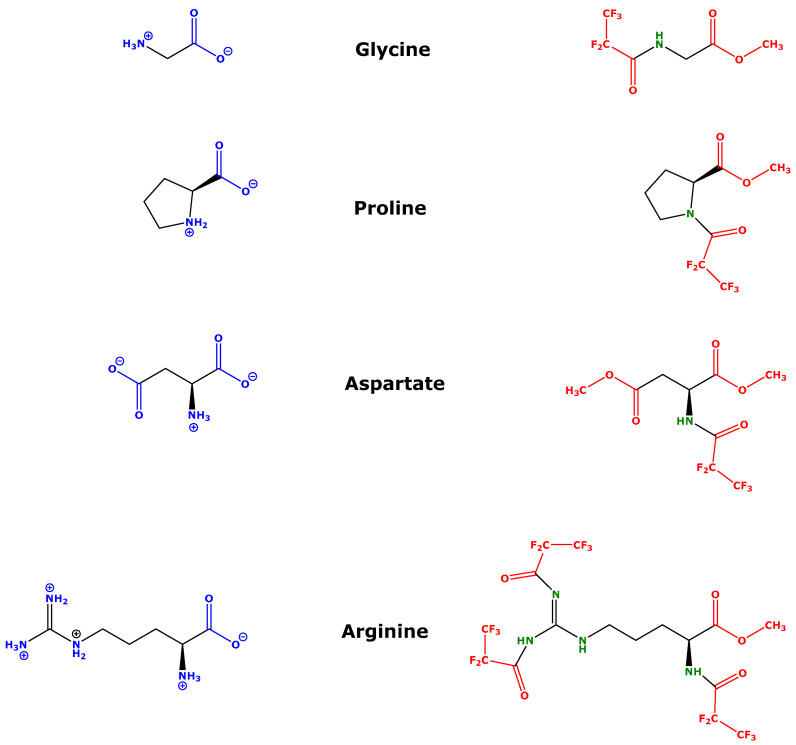
Chemical structures of four α-amino acids in their native form as they occur in aqueous buffered solutions (**left**) and in their derivatized forms (**right**), which are charge-free and soluble in and extractable into water-immiscible, GC-compatible organic solvents such as toluene. Blue indicates water-soluble parts and red indicates lipophilic parts of the amino acids. See also Figure 2.

Alpha-amino acids are carboxylic acids that contain at least one primary (NH_2_) or secondary (NH) amine group (Figure 1). They occur in biological samples in their free form, as well as residues in proteins. Amino acids are soluble in water and in water-miscible organic solvents such as methanol. In aqueous solutions, amino acids are ionized at any pH value due to their zwitterionic nature, with their carboxylic groups being deprotonated and their amine groups being protonated (Figure 1). Amino acids are not soluble in water-immiscible organic solvents such as toluene. Amino acids are generally not accessible to gas chromatography (GC)-based analysis because they are not volatile; they are thermally labile and would decompose to CO_2_, NH_3_, H_2_O and presumably to other species. Gas chromatography-mass spectrometry (GC-MS) analysis of amino acids requires their conversion to lipophilic derivatives that are extractable into GC-compatible, water-immiscible organic, volatile and thermally stable solvents such as toluene [1,2,3,4] (Figure 1).

**Figure 2:**
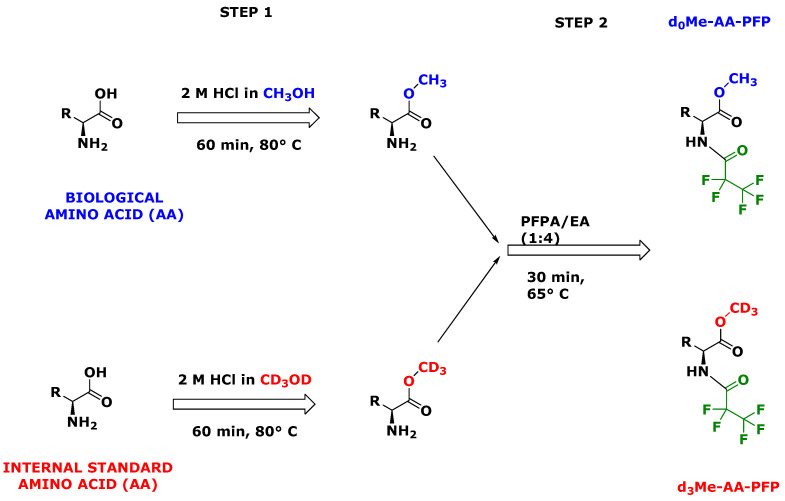
Two-step derivatization of alpha-amino acids ((NH_2_)R-COOH; R, residue of the side-chain) to their methyl ester (Me) pentafluoropropionyl (PFP) derivatives. In step 1, biological amino acids and synthetic amino acids (AA) for use as internal standards are converted separately to their unlabeled methyl esters (d_0_Me-AA) and their deuterium-labeled methyl esters (d_3_Me-AA). Subsequently, in step 2, d_0_Me-AA and d_3_Me-AA are combined and acylated with pentafluoropropionic anhydride (PFPA) in ethyl acetate (EA) to their d_0_Me-AA-PFP and d_3_Me-AA-PFP derivatives.

Amino acids (AA) can be specifically converted to their methyl esters (Me) by heating the samples in 2 M HCl in methanol (CH_3_OH) or deuterated methanol (CD_3_OD) [5]. The reaction products are unlabeled Me, i.e., d_0_Me-AA, and deuterated Me, i.e., d_3_Me-AA. Primary, secondary amine and imine groups of amino acids are subsequently acylated preferably by using perfluorated organic anhydrides such as pentafluoropropionic anhydride (PFPA) (Figure 2) [2,5]. This two-step derivatization procedure enables the in situ preparation of d_3_Me-AA for use as internal standards in quantitative GC-MS [6] (Figure 2). These derivatization procedures and the high long-term stability of such derivatives in toluene [7] allow for a high-throughput stable-isotope dilution GC-MS analysis of amino acids in biological samples such as human plasma in OMICS-format and at low costs. The utility of GC-MS for the high-throughput quantitative measurement of microbial metabolome has been reported [8].

In LC-MS-based metabolomics, quality control (QC) systems are essential for the assurance of analytical quality in biological systems [9,10,11,12,13,14,15,16,17]. In GC-MS-based methods for the measurements of polar metabolites such as nitrite (NO_2_^−^) and nitrate (NO_3_^−^) in biological samples, we developed and used QC systems [18]. The aim of the present work was to develop a QC system suitable for the GC-MS measurement of biological amino acids [6] that uses in situ preparation of d_3_Me-AA as internal standards [5]. The present work reports on the development, characterization and implementation of such a QC system for the high-throughput quantitative determination of amino acids in human plasma samples in clinical settings in an OMICS-like fashion. The utility of the d_3_Me-AA-PFP derivatives based on the extent of their peak area values and the ratio of and the difference in their retention times with respect to the d_0_Me-AA-PFP derivatives, i.e., the isotope effect, were also investigated as additional QC parameters.

## 2. Materials and Methods

### 2.1. Chemicals and Reagents

All amino acids, tetradeuterated methanol (CD_3_OD, 99% at ^2^H) and pentafluoropropionic anhydride (PFPA) were supplied by Sigma-Aldrich (Steinheim, Germany). Methanol (CH_3_OH) was obtained from Chemsolute (Renningen, Germany). Hydrochloric acid (37 wt%) was purchased from Baker (Deventer, The Netherlands) and was used to prepare the esterification reagents (2 M HCl in CH_3_OH or CD_3_OD). Ethyl acetate (EA) was obtained from Merck (Darmstadt, Germany) and was used to prepare the acylation reagent (PFPA in EA). Glassware for GC-MS, i.e., 1.5 mL autosampler vials and 0.2 mL microvials, were purchased from Macherey-Nagel (Düren, Germany). Deionized water (18.2 MΩ cm^−1^) was prepared using a Milli-Q device (Millipore Purification System, Merck, Darmstadt, Germany).

**Figure 3:**
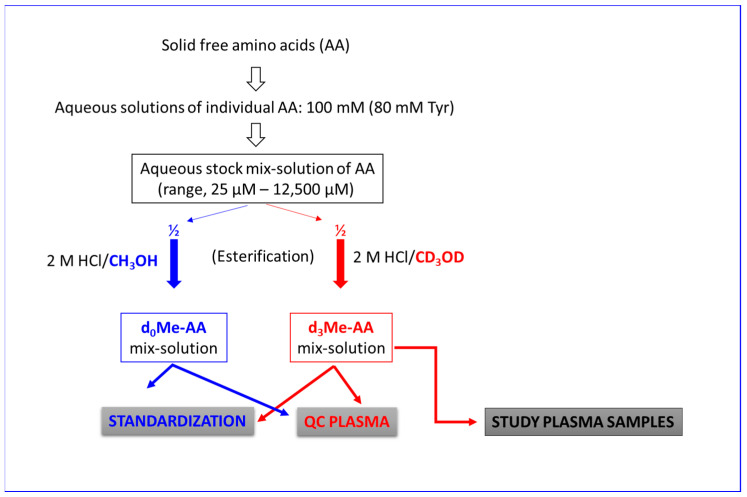
Simplified schematic of the sample preparation procedures used in the study in the standardization experiment, in the quality control (QC) plasma samples and in the analysis of amino acids (AA) in the study human samples. The procedures including those that follow the spiking of the AA are described in detail in the Materials and Methods section. d_0_Me-AA, unlabeled methyl esters (Me) of AA; d_3_Me-AA, deuterium-labeled methyl esters of AA. ½, half of the sample. “Solid free amino acids” means salts or bases of free amino acids from commercial sources.

Separate stock solutions of AA were prepared by dissolving accurately weighed amounts of the commercially available AA and their metabolites in deionized water (Figure 3). The concentrations of the AA in their stock solutions were 100 mM except for tyrosine, which was 80 mM. Aliquots (10 µL to 125 µL) of these solutions were transferred into a 1.8 mL glass vial. Deionized water was added to reach a total final volume of 1000 µL. The concentrations of the individual AA in this solution ranged between 25 µM (for ADMA) and 12.5 mM (for Glu/Gln) (Table 1). This stock AA solution was used in the present study.

For the preparation of unlabeled methyl esters of AA (d_0_Me-AA) and deuterium-labeled methyl esters of AA (d_3_Me-AA), two derivatization reagents were used (Figure 2). The esterification reagent was 2 M HCl in CD_3_OD for d_3_Me-AA and 2 M HCl in CH_3_OH for d_0_Me-AA. The acylation reagent PFPA-EA was prepared daily by diluting pure PFPA in EA (1:4, *v*/*v*).

### 2.2. Standardization of Amino Acid Concentrations in Deionized Water

Both commercially available and homemade isotopologues of physiological substances such as amino acids [5,19] and drugs such as acetazolamide [20] require mutual standardization with unlabeled reference compounds. Standardization has not been sufficiently addressed in the literature thus far. In LC-MS/MS, the term calibration is often used instead of the term standardization [21]. In the present work, an experiment was performed for the standardization of the concentrations of the AA in their aqueous solutions as described below (see also Figure 3).

The standardization (STD) samples were divided into four groups each consisting of two samples of equal AA concentrations: STD1.1, STD1.2; STD2.1, STD2.2; STD3.1, STD3.2; and STD4.1, STD4.2. After the first derivatization step, all STD samples were spiked with 10 µL aliquots of the solution d_3_Me-AA. STD1.1 and STD1.2. samples were not spiked with the solution d_0_Me-AA. STD2.1 and STD2.2 were spiked with 2.5 µL of solution d_0_Me-AA, STD3.1 and STD3.2 with 5.0 µL of solution d_0_Me-AA and STD4.1 and STD4.2 were spiked with 10 µL of solution d_0_Me-AA. The final nominal concentrations of the IS d_3_Me-AA and of d_0_Me-AA in the STD samples are listed in Table 1.

All STD samples (i.e., mixtures of d_0_Me-AA and d_3_Me-AA) were evaporated to dryness under a stream of nitrogen gas. Subsequently, all STD samples were treated with 100 µL aliquots of PFPA-EA (1:4, *v*/*v*); the samples were sealed and heated for 30 min at 65 °C. After the second derivatization step, the STD samples were evaporated to dryness under a stream of nitrogen gas. The residues were reconstituted in 200 µL borate buffer (0.4 M, pH 8.5) and immediately mixed by vortexing for 1 min with 200 µL toluene. Subsequently, the samples were centrifuged (5 min, 3400× *g*, 4 °C), and 150 µL aliquots of the supernatants (toluene phase) were transferred into autosampler glass vials that were equipped with microinserts. The samples were sealed and subjected to GC-MS analysis. Loss of d_0_Me-AA, d_3_Me-AA, d_0_Me-AA-PFP and d_3_Me-AA-PFP during the evaporation steps is possible but has not been investigated thus far. The internal standards are expected to compensate for any losses of d_0_Me-AA and d_0_Me-AA-PFP.

### 2.3. Preparation and GC-MS Analysis of Human Plasma Quality Control Samples

Pooled plasma was generated from EDTA blood previously sent from healthy humans to the blood bank of the Hannover Medical School and was used for QC. The QC plasma was portioned in 200 µL aliquots that were stored at −20 °C until use.

A schematic of the procedure is shown in Figure 3. For each run of study samples, a 200 µL aliquot of the QC plasma was thawed on ice, and 1000 µL of an ice-cold solution of 2 M HCl in CH_3_OH was added. The samples were centrifuged (5 min, 3400× *g*, 4 °C) to remove precipitated plasma proteins. Eight 40 µL aliquots of the clear supernatant were transferred into 1.8 mL autosampler glass vials. Samples were evaporated to dryness under a stream of nitrogen gas. Each study sample was treated with 100 µL 2 M HCl in CH_3_OH, closed tightly and heated at 80 °C for 1 h to prepare the unlabeled methyl esters of the endogenous AA in the QC plasma samples (d_0_Me-AA).

In parallel, two 40 µL aliquots of a stock solution of a mixture of synthetic AA were transferred into autosampler glass vials; the samples were evaporated to dryness under a nitrogen stream. The residue of one sample was reconstituted in 1000 µL of 2 M HCl in CD_3_OD; the sample was sealed tightly and heated at 80 °C for 1 h to prepare the deuterated methyl esters of the synthetic AA (d_3_Me-AA) for use as IS.

The residue of the second 40 µL aliquot was treated with 1000 µL 2 M HCl in CH_3_OH, closed tightly and heated at 80 °C for 1 h to prepare the unlabeled methyl esters (d_0_Me-AA) of the endogenous AA d_0_Me-AA in the QC plasma samples.

The eight QC samples were divided into four groups each consisting of two samples of the same concentrations of endogenous plasma: QC1.1, QC1.2; QC2.1, QC2.2; QC3.1, QC3.2; and QC4.1, QC4.2. After the first derivatization step, all QC samples were spiked with 10 µL aliquots of the solution d_3_Me-AA (IS). QC1.1 and QC1.2. samples were not spiked with the solution d_0_Me-AA. QC2.1 and QC2.2 were spiked with 2.5 µL of the solution d_0_Me-AA, QC3.1 and QC3.2 with 5.0 µL of the solution d_0_Me-AA and QC4.1 and QC4.2 were spiked with 10 µL of the solution d_0_Me-AA. The final nominal concentrations of the IS and of the added AA in the QC plasma samples are listed in Table 1.

All QC samples were evaporated to dryness under a stream of nitrogen gas. Subsequently, all samples were treated with 100 µL aliquots of PFPA-EA (1:4, *v*/*v*). The samples were sealed and heated for 30 min at 65 °C. Thereafter, the samples were evaporated to dryness under a stream of nitrogen gas. The residues were reconstituted in 200 µL borate buffer (0.4 M, pH 8.5) and immediately mixed by vortexing for 1 min with 200 µL toluene. Subsequently, the samples were centrifuged, and 150 µL aliquots of the supernatants (toluene phase) were transferred into autosampler glass vials that were equipped with microinserts. The samples were sealed and subjected to GC-MS analysis.

The precision of GC-MS analysis was determined as relative standard deviation (RSD, %)/coefficient of variation (CV, %) in all QC samples, which were analyzed in duplicate. The accuracy was determined as recovery for added AA concentrations in the QC2, QC3 and QC4 samples using Formula (1). In this Formula, [AA]_m_ is the AA concentration measured (m) in QC2, QC3 and QC4; [AA]_b_ is the baseline (b) AA concentration measured in QC1; and [AA]_a_ is the concentration of the added (a) AA. Mean accuracy in terms of recovery was calculated by linear regression analysis between the measured AA concentrations in all QC samples (*y*) and the added AA concentrations (*x*). The *y*-axis intercept (a) of the regression equation *y* = a + b × *x* provides the mean basal AA concentrations [AA]_b_ in the QC plasma, and the slope (b) of the regression multiplied by 100 yields the mean recovery rate.

Recovery (%) = ([AA]_m_ − [AA]_b_)/([AA]_a_)×100 (1)

### 2.4. Procedure for the GC-MS Analysis of Amino Acids in Human Plasma Samples

The nutritional human study was registered on ClinicalTrials.gov (NCT04923555) and performed after approval by an ethical committee (COMITE DE PROTECTION DES PERSONNES NORD OUEST III CHU—Niveau 03—Porte 03-363—Avenue de la Côte de Nacre 14033 Caen Cedex 09). Blood was drawn via a catheter inserted into a superficial arm vein, and samples were collected in the fasting state and in the postprandial state. EDTA vacutainers were centrifuged (4 °C, 15 min, 1000× *g*) and plasma samples were portioned, immediately plunged into liquid nitrogen and stored at −80 °C for subsequent analyses.

A schematic of the procedure is shown in Figure 3. Study human plasma samples were analyzed as follows. Frozen plasma samples (100 µL, −20 °C) were thawed on ice and centrifuged (5 min, 3400× *g*, 4 °C). Aliquots (10 µL) of the supernatants were transferred into Eppendorf tubes and treated with 50 µL of ice-cold 2 M HCl in CH_3_OH. Precipitated proteins were removed by centrifugation (5 min, 3400× *g*, 4 °C). Aliquots (40 µL) of the supernatants were transferred into autosampler glass vials, and 100 µL 2 M HCl in CH_3_OH was added. After esterification and cooling to room temperature, 10 µL aliquots of a freshly prepared d_3_Me-AA solution A (IS) were added. The concentrations of the respective IS in the samples are listed in Table 1. Upon evaporation to dryness under a stream of nitrogen gas, the second derivatization step with PFPA-EA was performed. Then, the derivatives were extracted with toluene/borate buffer (200 µL, 200 µL) by vortexing for 1 min. Subsequently, the samples were centrifuged, and 150 µL aliquots of the supernatants were transferred into autosampler glass vials equipped with microinserts. The samples were sealed and subjected to GC-MS analysis.

### 2.5. Order of Analysis of the Study Human Plasma Samples and QC Samples

In total, 327 study human plasma samples and 64 QC human plasma samples were analyzed in eight runs on seven days by a single person. The numbers of the study samples per run were 36, 44, 47, 47, 48, 43, 47 and 19. The toluene extracts of the nutritional human study and QC samples were placed onto the autosampler that was kept at a constant room temperature (19 °C). In all runs, the sequence of analysis was first a toluene sample that was injected two times, then the study samples and finally the eight QC samples (order, QC1, QC2, QC3, QC4). Each GC-MS analysis took about 22 min, resulting in a total analysis time of 16 h to 20 h per run. GC-MS analyses were performed automatically overnight.

Each run of analysis started with the injection of 1 µL aliquots of a pure toluene sample in duplicate, of the study human plasma samples and of the QC samples. Between each injection, the 10 µL Hamilton syringe of the autosampler was rinsed four times with pure toluene. The same procedure was used for all runs until the completion of the analysis of all study human plasma samples. About 50 to 60 samples per day were worked out by a single person and analyzed by GC-MS overnight. During these analyses, no services or MS tuning were performed on the GC-MS apparatus. Solely toluene on the autosampler used for rinsing the Hamilton syringe was daily refreshed.

### 2.6. GC-MS Analyses

Analyses were performed on a GC-MS apparatus consisting of a single-stage quadrupole mass spectrometer model ISQ, a Trace 1210 series gas chromatograph and an AS1310 autosampler from ThermoFisher (Dreieich, Germany). A fused-silica capillary column Optima 17 (15 m length, 0.25 mm I.D., 0.25 µm film thickness) from Macherey-Nagel (Düren, Germany) was used. Aliquots (1 µL) of toluene extracts were injected in the splitless mode. After injection, the 10 µL Hamilton syringe of the autosampler was rinsed four times with toluene. The injector temperature was kept at 280 °C. Helium was used as the carrier gas at a constant flow rate of 1.0 mL/min. The oven temperature was held at 40 °C for 0.5 min and ramped to 210 °C at a rate of 15 °C/min and then to 320 °C at a rate of 35 °C/min. The interface and ion-source temperatures were set to 300 °C and 250 °C, respectively. The electron energy was 70 eV and the electron current was 50 µA. Methane (2.4 mL/min) was used as the reagent gas for negative-ion chemical ionization (NICI). Quantitation was performed in the selected-ion monitoring (SIM) mode (Table 1). It should be noted that during the esterification procedure, citrulline (Cit) is converted to ornithine (Orn), asparagine (Asn) to aspartate (Asp) and glutamine (Gln) to glutamate (Glu). By this GC-MS method, the sum of Cit and Orn (Orn/Cit), the sum of Asn and Asp (Asp/Asn) and the sum of Glu and Gln (Glu/Gln) is measured [6]. As the derivatives of leucine (Leu) and isoleucine (IlE) are not separated by this GC-MS method, the sum of Leu and Ile (Leu/Ile) is measured [6] (Table 1). We did not investigate the GC-MS behavior of *allo*-isoleucine and cannot exclude that this GC-MS method also determines this AA in addition to Leu and Ile.

The peak area (PA) values of AA and of the respective internal standards were calculated automatically by the GC-MS software (Xcalibur and Quan Browser). The concentration of an endogenous AA (C_AA_) was determined by multiplying the peak area ratio (PAR) of an endogenous amino acid (PA_AA_) to its internal standard amino acid (PA_IS_) by the known concentrations of the respective internal standards [IS] (Table 1).

PAR_AA_ = PA_AA_/PA_IS_ (2)

[AA] = PAR_AA_ × [IS] (3)

The H/D isotope effect (IE) was calculated by dividing the retention time of the protiated AA *t*_R(H)_ by the retention time of the deuterated AA *t*_R(D)_, i.e., the internal standard (Formula (4)). The difference between *t*_R(H)_ and *t*_R_(_D)_, i.e., δ_(H/D)_, was calculated by Formula (5) and multiplied by 60 to obtain the difference in units of s.

IE = *t*_R(H)_/*t*_R(D)_ (4)

δ_(H/D)_ = *t*_R(H)_ − *t*_R(D)_ (5)

### 2.7. Data Handling–Statistics

Data analyses were performed using GraphPad Prism 7 for Windows (GraphPad Software, San Diego, CA, USA). GraphPad Prism 7 was also used to calculate the area under the curve (AUC) values in receiver operating characteristic (ROC) analyses and to prepare the graphs. Data are presented as mean ± SD if not otherwise specified. Within all statistical analyses, a two-sided *p*-value of less than 0.05 was considered statistically significant.

## 3. Results

### 3.1. Standardization of Amino Acid Concentrations in Deionized Water Solutions

The results of the standardization experiments are summarized in Table 2. Linear regression analysis between the PAR_AA_ measured (*y*) and the nominal AA concentrations (*x*) resulted in straight lines (*r*^2^ ≥ 0.9940). The *y*-axis intercept values were close to zero indicating the absence of unlabeled AA in the AA solutions in deionized water. The precision (CV, %), by which the concentrations STD2, STD3 and STD4 were measured, is in acceptable ranges. The reciprocal value of the slope of the regression equation corresponds to the standardized concentrations of the IS [5,19,20] (Formula (6)). The molar ratio of nominal to standardized IS ranged between 0.75 for OH-Pro and 1.60 for Val (0.93 ± 0.19).

PAR_AA_ = PA_AA_/PA_IS_ = [AA]_b_ + 1/[IS] × [STD] = *y* = a + b × *x* (6)

### 3.2. Amino Acids in Quality Control and Study Plasma Samples

**Figure 4:**
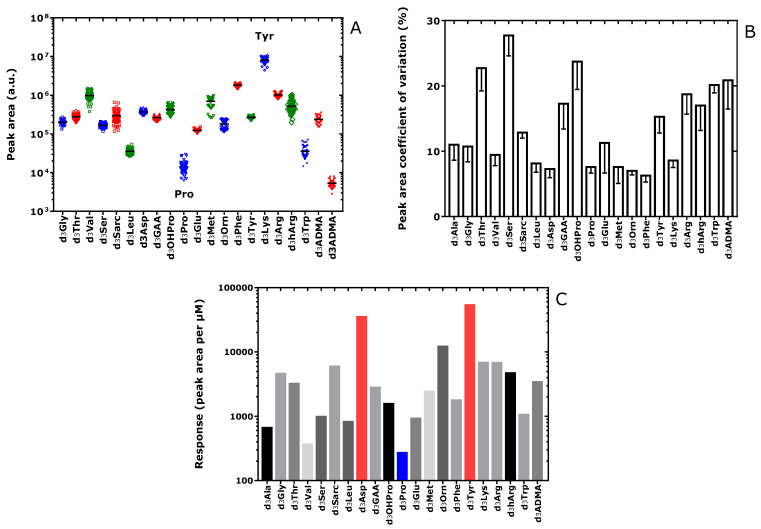
(**A**) Peak area values (a.u., arbitrary units), (**B**) coefficients of variation (%) of the peak area (mean—standard error of the mean) and (**C**) molar response of the peak area of the internal standards (trideutero-methyl esters (d_3_) of the amino acids in the plasma quality control (QC) samples as measured by GC-MS. Note the decadic logarithm scale on the *y*-axis in (**A**,**C**).

The PA values of the internal standards measured in the SIM mode in the QC plasma samples (Table S1, Figure 4A–C) and in the study plasma samples (Table S2) varied differently for each amino acid. The CV of the PA values ranged between 6% for Phe and 28% for Ser (Figure 4B). The PA values of the internal standards measured in QC samples were divided by the respective nominal concentrations of the internal standards used in the samples (see Table 1) in order to determine the molar GC-MS responses (Table S1, Figure 4). The highest molar response values were observed for Tyr and Asp/Asn. The lowest molar response values were obtained for Pro and Val. Both GC and MS factors are likely to have contributed to these differences. The largest contributions are presumably due to the abundant NICI of the Tyr and Asp/Asn derivatives (i.e., Tyr-d_3_Me-PFP and Asp/Asn-d_3_Me-PFP).

Table 3 summarizes the GC-MS results of the QC plasma samples. The *t*_R_ values of the d_0_Me-PFP derivatives ranged between 3.38 min (Ala) and 11.08 min (ADMA). The highest IE values were observed for Ser, Asp/Asn and Met. The largest δ_(H/D)_ values were observed for Met, Glu/Gln and Asp/Asn. Linear regression analysis between measured and added AA concentrations resulted in high linearity (*r*^2^ > 0.99). The *y*-axis intercept value provides the baseline concentrations of the AA, which ranged between 0.61 µM (for ADMA) and 985 µM (for Glu/Gln). The baseline concentrations of all AA measured in the pooled QC human sample are within normal ranges. The slope values of the regression straight lines are a measure of the mean recovery, which ranges between 79% for hArg and 125% for Glu/Gln. The mean precision of the QC human plasma samples ranged between 1.1% and 5.9% (Table 4).

In very few cases, peaks were observed from analyses of the toluene samples that were injected prior to the study samples within each run. In these analyses, the PA values of some AA such as Orn/Cit and Tyr amounted to less than 0.02% of the mean PA values of the internal standards. These observations suggest an almost complete absence of carryover in this GC-MS method.

Table S3 summarizes the results of the PA values of the internal standard in the study samples and in the co-processed QC samples. The ratio of the median PA values ranged between 0.17 and 1.55. The peak area ratio (PAR) of unlabeled to labeled AA ranged between 0.04 and 19.4 in the study and QC samples. The ratio of the mean PAR values in the study samples and in the QC samples ranged between 0.16 and 2.39.

**Figure 5:**
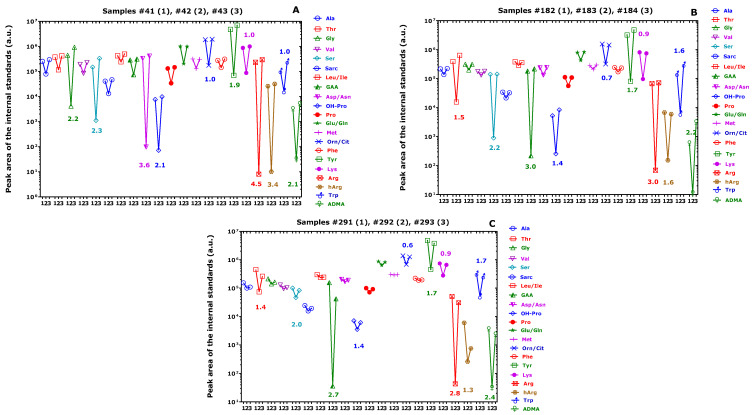
Plots of the peak ratio of the internal standards of the indicated amino acids in the study plasma samples (**A**) #41, #42, #43 (run 2); (**B**) #182, #183, #184 (run 5); and (**C**) #291, #292, #293 (run 7). Inserted numbers are the decadic logarithm of the ratios of the mean peak areas of samples 1 and 3 by the peak area of sample 2. In the term “123” on the *x*-axis, 1 indicates #41, #182 and #291; 2 indicates #42, #183 and #292; and 3 indicates #43, #184 and #293. Note the decadic logarithm on the *y*-axis.

In 3 out of 327 study plasma samples, we observed dramatic drops in the PA values of the internal standards (sample 2) as compared to the preceding (sample 1) and subsequent (sample 3) samples (Figure 5). The PA value decrease was observed in all AA but to a greatly varying order of magnitude ranging between a factor of 2 and a factor of 30,000. A possible explanation could be an injection of a small volume (<1 µL) from the toluene extracts of the affected samples. Another explanation could be impaired derivatization of the AA in affected study plasma samples. The greatest drops were found for GAA, Arg, hArg and ADMA. Such a drop in the PA of the internal standards was not observed in the QC plasma samples.

The corresponding values of the area under the receiver operating curves (ROC-AUC) for samples 1 and 2 were calculated from the data reported in Figure 5. They were determined to be (mean ± standard error of the mean) 0.864 ± 0.059 (*p* < 0.0001), 0.755 ± 0.074 (*p* = 0.0047) and 0.689 ± 0.081 (*p* = 0.0357). The ROC-AUC values of samples 1 and 3 did not differ (0.571 ± 0.090 (*p* = 0.428), 0.524 ± 0.090 (*p* = 0.792), 0.560 ± 0.090 (*p* = 0.505)). These results indicate that the PA values of the amino acids in samples 1 and 2 are statistically different, while those of samples 1 and 3 did not differ from each other.

Table 5 summarizes the GC-MS results of the study samples (range, *n* = 297–353) and QC samples (range, *n* = 54–64) for the analyzed amino acids with respect to *t*_R_(H), *t*_R_(D), IE and (δ(H/D) as measured during the eight runs of the study. IE and δ(H/D) values were close in the study of the QC samples and ranged between 1.002 and 1.006, and 0.84 s and 2.64 s. Concentrations of the AA measured by GC-MS in the study human plasma samples and their CV are listed in Table 6.

## 4. Discussion

The plasma concentrations of the AA measured in the study human plasma samples of the present work are all within normal ranges [21,22,23,24,25,26]. The occurrence of biological AA in wide concentration ranges in the presence of numerous other low-molecular-mass and high-molecular-mass physiological substances may represent a considerable analytical challenge, even for sophisticated instrumental techniques such as those based on mass spectrometry. Mass spectrometry (MS) is currently the sole analytical instrumental technique that enables the use of isotopically labeled amino acids as internal standards for accurate quantitative analysis.

Amino acids labeled with a sufficient number of stable isotopes, commonly ^2^H, ^13^C and ^15^N, with high isotopic purity are commercially available. As a practical and cost-saving alternative, we proposed the in situ preparation of trideutero-methyl esters of unlabeled commercially available AA [5]. Thus, individual AA and their mixtures are heated in 2 M HCl in CD_3_OD to generate specifically their trideutero-methyl esters (d_3_Me-AA) in high yield (about 85%) and high isotopic purity (Figure 2). The use of CD_3_OD of the highest commercially available isotopic purity is highly recommended. Separate performance of the esterification reaction in 2 M HCl in CH_3_OH results in the formation of the unlabeled AA methyl esters (d_0_Me-AA). Subsequent derivatization of the AA methyl esters with PFPA converts accessible amine, hydroxyl and sulfhydryl groups to their PFP acyl derivatives. Methyl ester pentafluoropropionyl derivatives of AA (d_0_Me-AA-PFP and d_3_Me-AA-PFP) are lipophilic, charge-free and stable in toluene extracts. They are best suitable for automated high-throughput quantitative analysis of AA in various biological samples including human plasma and urine by GC-MS and GC-MS/MS [5,6,7].

Implementation of quality control (QC) systems in analytical chemistry is indispensable to control and ensure the quality of all kinds of chemical and immunological analyses [9,10,11,12,13,14,15,16,17,18,26]. The present work proposes the use of a QC system for the targeted, stable-isotope dilution-based GC-MS measurement of AA in human plasma samples in clinical studies as d_0_Me-AA-PFP and d_3_Me-AA-PFP derivatives. This QC system was adapted to the de novo synthesis of d_3_Me-AA [5] and considers the physiological occurrence of AA [21,22,23,24,25,26], which deserves special handling unlike exogenous substances, notably, drugs [27,28]. In GC-MS- and LC-MS-based untargeted metabolomics, analytical errors may result, among others, from instrumental drifts, such as shifts in retention time (GC-related factors) and metabolite intensities (MS-related factors). These kinds of errors and the utility of so-called intra-study QC samples were recently reviewed and discussed in detail, and helpful recommendations were made [29].

The present work demonstrates that physiological AA can be measured quantitatively in human plasma samples by a targeted GC-MS method in an OMICS-like fashion. In total, in 8 runs, 353 study plasma samples and 64 QC plasma samples were analyzed for 21 amino acids within 7 working days by a single person. Study plasma samples were analyzed once, and QC plasma samples were analyzed in duplicate at four different AA concentrations. The selected concentrations of the AA spiked to the QC1, QC2, QC3 and QC4 samples were all in relevant ranges for human plasma. The concentrations of the in situ prepared internal standards added to the study, and QC plasma samples were also in relevant concentrations.

AA from the same stock solutions and their dilutions in deionized water were used both in the study human plasma samples and in the QC plasma samples. For this purpose, synthetic AA were esterified to their unlabeled methyl ester (d_0_Me-AA) and deuterium-labeled methyl ester (d_3_Me-AA). d_0_Me-AA were used at varying relevant concentrations. d_3_Me-AA were used as internal standards at fixed concentrations (d_3_Me-AA). This procedure requires cross-standardization of d_0_Me-AA and d_3_Me-AA in their aqueous solutions. The standardization of homemade and commercially available stable-isotope labeled analogs has been reported in quantitative GC-MS for numerous classes of analytes including eicosanoids and amino acids [30,31]. In the standardization experiment reported here, linear regression analysis between the PAR of d_0_Me-AA-PFP and d_3_Me-AA-PFP derivatives and the tested AA concentrations (STD1, STD2, STD3 and STD4) resulted in high linearity. The reciprocal of the slope values of the straight lines corresponds to the concentrations of the internal standards (Formula (7)). These values were used to standardize the concentrations of the internal standard d_3_Me-AA.

PAR_AA_ = PA_AA_/PA_IS_ = [AA]_b_ + **1/[IS]** × [STD] = *y* = a + **b** × *x* (7)

Study human plasma samples and QC samples of each run were worked out and analyzed by GC-MS in parallel. The PA values of the internal standards were comparable in the study human plasma samples and in the QC samples and they varied to a comparable degree. The normalized PA values, i.e., the PA values per µM amino acid, differed greatly between the AA (range, 271 for Pro to 53,404 for Phe). Despite greatly differing PA values (about 200-fold for Pro and Phe), the concentration of all AA analyzed in the present study was measured accurately and precisely as demonstrated by analyzing the QC human plasma samples. Mean recovery values ranged between 79% and 125%, whereas the precision (CV, %) was clearly below 20%. This is certainly due to the use of isotopically labeled AA, i.e., d_3_Me-AA, as internal standards.

Observed small changes in the retention times of Me-PFP derivatives of the AA are attributed to MS- and GC-related factors. No noteworthy variations were observed for IE and δ(H/D), suggesting that changes occurred to the same degree to the endogenous AA d_0_Me-AA-PFP and to their internal standards (d_3_Me-AA-PFP). IE and δ(H/D) may be suitable as additional parameters in QC systems, yet they are unlikely to replace the QC systems that are used to determine accuracy and precision in methods of quantitative analysis.

In very few cases of the study human plasma samples, we observed dramatic falls in the PA values of the internal standards. Recurrence of the complete analytical procedure of these samples resulted in PA values being in ranges close to the mean PA values. In targeted GC-MS metabolomic studies, the PA values of the internal standards, which are all added to study samples at their typical fixed concentrations, are not only suitable to quantify concentrations of endogenous AA, but they are also useful to detect potential analytical problems of known (for instance failed injection of toluene extracts) and unknown identity. Further investigations are needed to define the extent of drops of the PA of internal standards that still permit accurate analysis of AA in human plasma by GC-MS. The utility of the PA values of endogenous AA is limited in this context because their concentrations are unknown and variable.

## 5. Conclusions

GC-MS is suitable for the quantitative analysis of AA upon chemical derivatization to their methyl ester pentafluoropropionyl (d_0_Me-AA-PFP) derivatives. In situ preparation of deuterated methyl esters of AA (d_3_Me-AA) for use as internal standards in GC-MS is a convenient and cost-saving laboratory method. d_0_Me-AA-PFP and d_3_Me-AA-PFP derivatives are readily extractable into GC-compatible organic solvents such as toluene and possess long-term stability therein. This feature enables high-throughput quantitative measurement of biological AA. The QC system described in this work is OMICS-compatible and suitable for the QC measurement of AA in human plasma samples. Trideutero-methyl esters of AA are also useful for controlling the quality of the analytical performance of this GC-MS method in clinical settings. IE and δ(H/D) of d_0_Me-AA-PFP and d_3_Me-AA-PFP may be suitable as additional parameters in QC systems in GC-MS measurements of AA in human plasma samples in an OMICS-like fashion.

## Figures and Tables

**Table 1:** Nominal (theoretical) concentrations (in µM) of the amino acids (AA) and their respective internal standards (IS) used in the standardization (STD) experiment and in the quality control (QC) plasma samples, ions (*m*/*z*) of AA (d_0_Me-AA-PFP) and IS (d_3_Me-AA-PFP) monitored by selected-ion monitoring (SIM) in the GC-MS analyses using the listed dwell times and time windows. Leu/Ile, Asp/Asn, Glu/Gln and Orn/Cit represent the sum of the paired AA.

AA	STD1; QC1 (µM)	STD2; QC2 (µM)	STD3; QC3 (µM)	STD4; QC4 (µM)	IS (µM)	Stock Solution (µM)	*m*/*z* of AA	*m*/*z* of IS	Dwell Time (ms)	Time Window (min)
Ala	0	75	150	300	200	5000	229	232	100	3.20
Thr	0	15	30	60	40	1000	259	262	50	3.65
Gly	0	75	150	300	200	5000	215	218	50	3.65
Val	0	112	224	448	300	7500	257	260	50	3.65
Ser	0	75	150	300	200	5000	207	210	50	3.65
Sarc	0	1.5	3.0	6.0	4	100	229	232	50	4.32
Leu/Ile	0	112	224	448	300	7500	271	274	100	5.10
GAA	0	1.9	3.8	7.6	50	125	383	386	50	5.85
Asp/Asn	0	37	74	148	100	2500	287	293	50	5.85
OH-Pro	0	22.5	45	90	6	1500	397	400	50	5.85
Pro	0	112	224	448	30	7500	255	258	100	6.52
Glu/Gln	0	187	375	750	500	1250	301	307	100	7.10
Met	0	19	38	76	50	12,500	289	292	100	7.10
Orn/Cit	0	37	74	148	100	2500	418	421	50	7.80
Phe	0	37	74	148	100	2500	305	308	50	7.80
Tyr	0	37	74	148	100	2500	233	236	100	8.35
Lys	0	37	74	148	100	2500	432	435	50	8.80
Arg	0	19	38	76	50	1250	586	589	50	8.80
hArg	0	1.9	3.8	7.6	5	125	600	603	100	9.75
Trp	0	56	112	224	150	3750	233	236	50	10.40
ADMA	0	0.37	0.74	1.48	1.0	25	634	637	100	10.40

**Table 2:** Results of the standardization experiment of the amino acids (AA) and the respective internal standards (IS) in deionized water solutions measured as d_0_Me-AA-PFP and d_3_Me-AA-PFP derivatives, respectively, by GC-MS in the selected-ion monitoring (SIM) mode. AA were used at the indicated nominal concentrations (STD1, STD2, STD3 and STD4). [IS]_nom_, nominal concentrations of the internal standards; [IS]_std_, standardized concentrations of the internal standards.

AA	STD1 (µM)	STD2 (µM)	STD3 (µM)	STD4 (µM)	[IS]_nom_ (µM)	Regression Equation (*y*= a + b × *x*) (*y* = PAR, *x* = [STD])	[IS]_std_ (µM)	[IS]_nom_ /[IS]_std_
Ala	0	75	150	300	200	*y* = −0.0007 + 0.0043 × *x*, *r*^2^ = 0.9995	232	0.86
(CV, %)	7.7	2.9	1.7	2.5				
Thr	0	15	30	60	40	*y* = −0.0067 + 0.03187 × *x*, *r*^2^ = 0.9999	31	1.29
(CV, %)	30	5.7	2.8	2.2				
Gly	0	75	150	300	200	*y* = −0.00024 + 0.0041 × *x*, *r*^2^ = 0.9999	243	0.82
(CV, %)	30	6.1	3.3	2.6				
Val	0	112	224	448	300	*y* = −0.02417 + 0.0530 × *x*, *r*^2^ = 0.9993	188	1.60
(CV, %)	53	5.0	2.4	4.6				
Ser	0	75	150	300	200	*y* = 0.01259 + 0.00466 × *x*, *r*^2^ = 0.9999	215	0.93
(CV, %)	28	4.5	3.6	2.7				
Sarc	0	1.5	3.0	6.0	4	*y* = 0.00472 + 0.2022 × *x*, *r*^2^ = 1.0000	5	0.80
(CV, %)	39	2.0	2.4	8.0				
Leu/Ile	0	112	224	448	300	*y* = 0.02317 + 0.00259 × *x*, *r*^2^ = 0.9998	386	0.78
(CV, %)	19	4.7	1.7	1.9				
GAA	0	1.9	3.8	7.6	50	*y* = 0.01701 + 0.01816 × *x*, *r*^2^ = 0.9974	55	0.91
(CV, %)	30	65	11.3	38				
Asp/Asn	0	37	74	148	100	*y* = 0.02115 + 0.00982 × *x*, *r*^2^ = 0.9989	102	0.98
(CV, %)	0	4.7	2.6	2.9				
OH-Pro	0	22.5	45	90	6	*y* = 0.02633 + 0.1322 × *x*, *r*^2^ = 0.9999	8	0.75
(CV, %)	0	4.8	4.9	9.2				
Pro	0	112	224	448	30	*y* = 0.0845 + 0.02901 × *x*, *r*^2^ = 0.9955	35	0.86
(CV, %)	17.1	5.7	3.3	4.4				
Glu/Gln	0	187	375	750	500	*y* = 0.04569 + 0.00192 × *x*, *r*^2^ = 0.9947	520	0.96
(CV, %)	0	6.3	2.2	2.8				
Met	0	19	38	76	50	*y* = 0.0792 + 0.0158 × *x*, *r*^2^ = 0.9940	63	0.79
(CV, %)	7.8	2.6	1.9	4.7				
Orn/Cit	0	37	74	148	100	*y* = −0.0131 + 0.00957 × *x*, *r*^2^ = 0.9995	105	0.95
(CV, %)	53	5.6	2.4	2.3				
Phe	0	37	74	148	100	*y* = −0.0044 + 0.00884 × *x*, *r*^2^ = 0.9997	113	0.88
(CV, %)	11.1	5.0	1.6	2.4				
Tyr	0	37	74	148	100	*y* = −0.0076 + 0.00789 × *x*, *r*^2^ = 0.9996	127	0.79
(CV, %)	28.4	5.5	2.7	1.8				
Lys	0	37	74	148	100	*y* = 0.0472 + 0.00865 × *x*, *r*^2^ = 0.9931	116	0.86
(CV, %)	44	5.3	2.3	3.0				
Arg	0	19	38	76	50	*y* = 0.0104 + 0.01776 × *x*, *r*^2^ = 0.9979	56	0.89
(CV, %)	28	12.3	13.2	9.1				
hArg	0	1.9	3.8	7.6	5	*y* = 0.0156 + 0.2167 × *x*, *r*^2^ = 0.9982	4.6	1.09
(CV, %)	28.4	10.5	17.6	6.6				
Trp	0	56	112	224	150	*y* = −0.0036 + 0.0056 × *x*, *r*^2^ = 0.9998	179	0.84
(CV, %)	38.5	6.1	4.2	4.1				
ADMA	0	0.37	0.74	1.48	1.0	*y* = 0.0558 + 0.8995 × *x*, *r*^2^ = 0.9998	1.1	0.91
(CV, %)	74.2	5.5	9.9	7.3				

**Table 3:** Retention times (*t*_R_) of the unlabeled amino acid methyl ester pentafluoropropionyl derivatives (d_0_Me-PFP), isotope effect (IE), difference (δ_(H/D)_) in *t*_R_ of unlabeled and deuterium labeled (d_3_Me-PFP) derivatives and regression equation (*y* = a + b × *x*) observed in the GC-MS analyses in the selected-ion monitoring (SIM) mode of the indicated amino acids (AA) in the QC plasma samples. Linear regression analysis between measured (*y*) and added (*x*) AA concentration was performed.

AA	*t*_R_	IE	δ_(H/D)_	Regression Equation			
	min (CV, %)	(CV, %)	(s)	*y*-axis Intercept (a)	Slope (b)	*r* ^2^	[IS]_std_ (µM)
Ala	3.382 (0.51)	1.005 (0.15)	0.96	430	0.91	0.9980	232
Thr	3.799 (0.23)	1.004 (0.15)	0.90	196	0.89	0.9968	31
Gly	3.794 (0.33)	1.005 (0.07)	1.22	247	0.91	0.9982	243
Val	4.017 (0.22)	1.005 (0.13)	1.17	367	0.92	0.9992	188
Ser	4.139 (0.16)	1.006 (0.15)	1.37	149	0.95	0.9990	215
Sarc	4.491 (0.17)	1.005 (0.08)	1.28	1.49	0.94	0.9984	5
Leu/Ile	4.644 (0.14)	1.004 (0.07)	1.17	196	0.96	0.9996	386
GAA	6.258 (0.09)	1.004 (0.08)	1.61	6.93	0.87	0.9986	55
Asp/Asn	6.200 (0.05)	1.006 (0.04)	2.40	60	0.99	0.9995	102
OH-Pro	6.400 (0.03)	1.003 (0.03)	1.18	10.1	1.08	0.9976	8
Pro	6.602 (0.06)	1.003 (0.05)	1.28	214	1.01	0.9998	35
Glu/Gln	7.383 (0.06)	1.005 (0.05)	2.42	985	1.25	0.9949	520
Met	7.393 (0.06)	1.006 (0.06)	2.56	86	0.97	0.9999	63
Orn/Cit	8.099 (0.07)	1.002 (0.00)	1.20	179	0.96	0.9988	105
Phe	8.154 (0.06)	1.002 (0.00)	1.20	78	0.99	0.9996	113
Tyr	8.578 (0.06)	1.002 (0.04)	1.14	96	0.92	0.9995	127
Lys	9.002 (0.07)	1.002 (0.05)	1.22	181	1.01	0.9995	116
Arg	9.230 (0.05)	1.002 (0.04)	1.20	62	0.86	0.9992	56
hArg	10.03 (0.20)	1.003 (0.06)	1.61	1.58	0.79	0.9997	4.6
Trp	10.92 (0.04)	1.002 (0.04)	1.58	31	1.23	0.9993	179
ADMA	11.08 (0.06)	1.002 (0.03)	1.12	0.609	0.80	0.9924	1.1

**Table 4:** Precision (coefficient of variation, %) of the measurement of the listed amino acids in the QC human plasma samples QC1, QC2, QC3 and QC4. Data are given as means with CV in parentheses.

AA	QC1	QC2	QC3	QC4	Mean QC
Ala	4.12 (3.95)	5.94 (3.58)	3.61 (3.44)	3.64 (3.94)	4.33 (1.10)
Thr	4.51 (4.72)	2.59 (2.04)	1.74 (1.69)	3.33 (3.94)	3.04 (1.17)
Gly	1.89 (1.16)	3.48 (2.21)	2.34 (1.57)	1.60 (1.69)	2.33 (0.83)
Val	2.90 (1.87)	2.94 (1.54)	2.79 (2.46)	2.39 (2.12)	2.75 (0.25)
Ser	2.04 (1.54)	2.10 (1.57)	3.04 (1.84)	1.82 (1.82)	2.25 (0.54)
Sarc	1.59 (1.17)	4.29 (3.28)	4.00 (2.69)	1.98 (1.89)	2.96 (1.37)
Leu/Ile	2.56 (1.79)	3.43 (1.71)	2.34 (2.19)	1.25 (0.59)	2.40 (0.90)
GAA	1.53 (1.19)	3.45 (1.74)	3.44 (3.66)	2.08 (1.75)	2.63 (0.97)
Asp/Asn	1.91 (1.00)	2.44 (1.86)	1.72 (2.35)	3.18 (3.07)	2.31 (0.65)
OH-Pro	5.39 (9.04)	3.96 (3.74)	3.70 (3.66)	2.78 (1.05)	3.96 (1.08)
Pro	3.16 (2.15)	4.64 (2.44)	3.87 (2.67)	2.97 (2.36)	3.61 (0.67)
Glu/Gln	4.28 (2.69)	3.41 (3.25)	4.87 (4.71)	2.99 (3.33)	3.88 (0.85)
Met	3.69 (6.00)	1.77 (2.02)	2.66 (2.27)	1.90 (1.50)	2.50 (0.88)
Orn/Cit	2.07 (2.03)	4.00 (2.90)	2.98 (3.26)	1.22 (1.34)	2.57 (1.19)
Phe	1.92 (1.20)	2.85 (1.67)	3.36 (3.07)	2.17 (1.54)	2.58 (0.66)
Tyr	3.06 (2.72)	3.97 (3.18)	3.43 (2.38)	1.39 (1.15)	2.96 (1.11)
Lys	2.88 (1.19)	2.12 (1.70)	1.93 (1.22)	1.32 (0.81)	2.06 (0.64)
Arg	2.17 (1.62)	3.57 (2.32)	3.35 (3.07)	1.64 (0.73)	2.68 (0.93)
hArg	1.53 (1.55)	1.91 (1.89)	3.24 (3.97)	1.07 (0.94)	1.94 (0.93)
Trp	2.44 (1.85)	3.68 (1.99)	2.99 (3.20)	2.65 (1.24)	2.94 (0.54)
ADMA	1.79 (0.73)	2.30 (1.10)	3.23 (3.84)	1.22 (1.04)	2.13 (0.86)
Mean QC	2.73 (1.10)	3.27 (1.02)	3.08 (0.78)	2.12 (0.79)	

**Table 5:** Results of the GC-MS analysis of the indicated amino acids in (A) study human plasma samples and in (B) quality control plasma samples as mean with the coefficients of variation being reported in parentheses. Reported are the retention times values of the unlabeled *t*_R_(H) and of the deuterium-labeled *t*_R_(D) methyl esters, the isotope effect (IE) values and the differences of the retention times (δ(H/D). The number of samples considered is given below the respective results. The number of analyses for IE and δ(H/D) is reported for IE ≥ 1.000 and δ(H/D) ≥ 0 and ≤2-x mean.

AA	*t*_R_(H) (min)	*t*_R_(D) (min)	IE	δ(H/D) (s)	*t*_R_(H) (min)	*t*_R_(D) (min)	IE	δ(H/D) (s)
	(A) Study Samples				(B) Quality Control Samples			
Ala	3.369 (0.5)	3.355 (0.6)	1.005 (0.2)	0.92 (44)	3.372 (0.5)	3.36 (0.7)	1.004 (0.3)	0.90 (56)
(*n*)	353	352	340	340	64	62	59	59
Thr	3.790 (0.3)	3.773 (0.5)	1.004 (0.3)	0.98 (77)	3.790 (0.3)	3.776 (0.3)	1.004 (0.2)	0.84 (58)
(*n*)	353	353	349	349	64	62	62	62
Gly	3.787 (0.3)	3.767 (0.5)	1.006 (0.2)	1.34 (27)	3.788 (0.3)	3.768 (0.6)	1.006 (0.2)	1.35 (30)
(*n*)	353	353	341	341	64	62	60	60
Val	4.007 (0.3)	3.984 (0.5)	1.005 (0.2)	1.23 (33)	4.007 (0.3)	3.983 (0.7)	1.006 (0.7)	1.43 (90)
(*n*)	353	352	344	344	64	62	62	62
Ser	4.128 (0.2)	4.107 (0.4)	1.005 (0.2)	1.17 (35)	4.128 (0.2)	4.105 (0.4)	1.006 (0.4)	1.39 (72)
(*n*)	353	353	344	323	64	61	61	54
Sarc	4.485 (0.2)	4.463 (0.3)	1.005 (0.1)	1.26 (19)	4.484 (0.2)	4.461 (0.3)	1.005 (0.3)	1.40 (59)
(*n*)	353	352	344	344	64	62	62	62
Leu/Ile	4.634 (0.2)	4.614 (0.3)	1.004 (0.1)	1.17 (25)	4.632 (0.2)	4.611 (0.3)	1.004 (0.2)	1.24 (49)
(*n*)	353	353	345	345	64	61	61	61
GAA	6.285 (0.1)	6.255 (0.1)	1.006 (0.1)	2.34 (50)	6.250 (0.1)	6.229 (0.1)	1.003 (0.1)	1.28 (22)
(*n*)	352	352	297	297	63	61	59	59
Asp/Asn	6.191 (0.1)	6.150 (0.1)	1.007 (0.1)	2.49 (16)	6.187 (0.1)	6.146 (0.1)	1.007 (0.1)	2.45 (18)
(*n*)	353	353	351	351	64	62	62	62
OH-Pro	6.393 (0.1)	6.375 (0.1)	1.003 (0.1)	1.11 (26)	6.400 (0.1)	6.381 (0.1)	1.003 (0.1)	1.19 (23)
(*n*)	353	353	350	350	64	62	61	61
Pro	6.594 (0.1)	6.574 (0.1)	1.003 (0.1)	1.23 (21)	6.593 (0.1)	6.573 (0.1)	1.003 (0.1)	1.21 (21)
(*n*)	353	352	347	347	64	62	61	61
Glu/Gln	7.372 (0.1)	7.332 (0.2)	1.006 (0.1)	2.44 (15)	7.373 (0.1)	7.329 (0.2)	1.006 (0.1)	2.64 (24)
(*n*)	353	352	352	352	64	62	62	62
Met	7.380 (0.1)	7.340 (0.1)	1.005 (0.1)	2.42 (16)	7.383 (0.1)	7.342 (0.2)	1.006 (0.2)	2.46 (31)
(*n*)	353	352	352	352	64	62	62	62
Orn/Cit	8.086 (0.1)	8.067 (0.2)	1.002 (0.1)	1.12 (19)	8.108 (0.2)	8.088 (0.2)	1.003 (0.1)	1.22 (24)
(*n*)	353	352	351	351	64	62	62	62
Phe	8.144 (0.1)	8.123 (0.1)	1.003 (0.1)	1.27 (22)	8.143 (0.1)	8.124 (0.1)	1.003 (0.1)	1.26 (21)
(*n*)	353	352	345	345	64	62	60	60
Tyr	8.566 (0.2)	8.548 (0.1)	1.002 (0.1)	1.13 (22)	8.581 (0.2)	8.562 (0.1)	1.002 (0.1)	1.15 (31)
(*n*)	353	353	350	350	63	62	61	61
Lys	8.988 (0.1)	8.970 (0.1)	1.002 (0.1)	1.06 (30)	8.995 (0.1)	8.977 (0.1)	1.002 (0.1)	1.11 (32)
(*n*)	353	353	351	351	64	62	62	62
Arg	9.284 (1.0)	9.255 (0.8)	1.003 (0.2)	1.68 (54)	9.219 (0.1)	9.206 (0.9)	1.002 (0.1)	1.00 (34)
(*n*)	353	350	324	324	63	62	59	60
hArg	10.13 (1.0)	10.11 (1.0)	1.003 (0.2)	1.80 (55)	10.03 (0.3)	10.01 (0.4)	1.003 (0.1)	1.66 (35)
(*n*)	351	347	314	310	64	61	59	59
Trp	10.91 (0.1)	10.89 (0.1)	1.002 (0.1)	1.42 (24)	10.91 (0.1)	10.89 (0.1)	1.002 (0.1)	1.41 (28)
(*n*)	353	352	348	348	64	62	62	62
ADMA	11.07 (0.1)	11.06 (0.1)	1.002 (0.1	1.10 (27)	11.07 (0.1)	11.05 (0.1)	1.002 (0.1	1.08 (25)
(*n*)	352	352	350	350	63	62	60	60

**Table 6:** Concentrations (µM) of the amino acids measured by GC-MS in the study human plasma samples (*n* = 337) and their coefficients of variation.

AA	Median	25% Percentile	75% Percentile	CV (%)
Ala	379	326	442	22
Gly	289	220	472	61
Thr	149	127	177	25
Val	437	351	529	31
Ser	121	107	143	37
Sar	10.7	8.8	12.2	29
Leu/Ile	217	177	307	40
Asp/Asn	54.3	46.7	61.6	22
GAA	6.24	5.4	7.2	47
OH-Pro	6.94	5.53	8.7	38
Pro	227	171	299	40
Gln/Glu	811	741	902	16
Met	70.2	65.5	75.2	11
Orn/Cit	87.9	73.1	106	26
Phe	71.6	61.0	84.9	24
Tyr	87.1	70.2	115	35
Lys	212	188	249	26
Arg	93.5	79.1	110.2	35
hArg	1.94	1.57	2.52	139
Trp	43.2	36.5	51.4	27
ADMA	0.57	0.48	0.64	25

## Data Availability

Data sharing is not applicable to this article.

## Supplementary Materials

The following supporting information can be downloaded at: https://www.mdpi.com/article/10.3390/metabo13090986/s1, Table S1: Mean peak area (PA) values (in arbitrary units) of the amino acids, coefficient of variation (CV, %) values and molar responses of the internal standards in the quality control (QC) samples within seven runs as analyzed by GC-MS. Table S2: Mean peak area (PA) values (in arbitrary units) of the amino acids and coefficient of variation (CV, %) values in the study samples within eight runs as analyzed by GC-MS. Table S3: Median peak area (PA) values of the internal standards (IS) from the GC-MS analyses of the study samples (A) and the plasma quality control (B) samples; peak area ratio (PAR) range in the study samples (C) and QC samples (D); and mean of the PAR in (C) and (D). n.s., not significant.
