# Supplementary material for: Quality Control in Targeted GC-MS for Amino Acid-OMICS
Source: Metabolites. 2023 Aug 31;13(9):986. doi: 10.3390/metabo13090986 (PMC10536693; doi:10.3390/metabo13090986)
Supplement: Supplementary file 1 [file metabolites-13-00986-s001.zip › metabolites-2577063-supplementary.pdf]

Supplementary Materials

Quality Control in Targeted GC-MS for Amino Acid-OMICS

Dimitrios Tsikas [1,*] and Bibiana Beckmann[1]

Institute of Toxicology, Core Unit Proteomics, Hannover Medical School, 30623 Hannover, Germany

Correspondence: tsikas.dimitros@mh-hannover.de

Table S1. Mean peak area (PA) values (in arbitrary units) of the amino acids, coefficient of variation (CV, %) values, and molar responses of the internal standards in the quality control (QC) samples within 7 runs as analyzed by GC-MS.

**AA**	Run 6		Run 5		Run 4		Run 3		Run 7		Run 2		Run 1		Response
	PA	CV	PA	CV	PA	CV	PA	CV	PA	CV	PA	CV	PA	CV	PA/μM
Ala	171748	11	184314	4	215958	6	222085	7	182990	10	199818	18	215670	21	663
Thr	240733	7	252543	5	305145	6	311380	9	254446	9	250143	19	314197	20	4592
Gly	697096	33	883970	22	1163932	12	1114381	16	811854	14	1010628	27	1074338	35	3217
Val	144322	9	161464	4	182309	5	174616	9	150682	9	165894	14	174592	16	366
Ser	203413	34	248212	28	342053	18	327067	18	224256	23	274700	38	447332	35	984
Sarc	33447	11	31161	11	42166	10	41387	16	31773	14	33144	15	37253	13	5960
Leu	334836	5	347816	5	393018	5	379154	9	340234	7	379309	13	410224	13	821
GAA	248293	4	260178	5	283128	5	262639	11	242627	5	252389	13	288098	8	2802
Asp/Asn	371376	9	316386	10	478998	20	478634	12	356095	16	442041	15	498433	39	34,997
OH-Pro	10402	31	11007	25	13970	16	13670	23	11863	6	18510	23	19375	42	1568
Pro	119241	6	120950	6	130775	5	122558	11	118397	7	117399	11	124932	7	271
Glu/Gln	748979	6	823146	6	895407	5	734504	9	766373	7	507096	7	393016	39	927
Met	199307	7	229249	5	237085	4	192406	2	128098	6	136111	7	152170	22	2427
Orn/Cit	1646531	5	1883747	7	1931475	8	1735854	6	1836783	6	1764153	10	1918661	7	12,112
Phe	258064	4	255823	5	278641	5	265624	9	254614	5	272079	11	283433	5	1779
Tyr	6808676	20	7750608	21	8614748	10	8534720	10	7804658	8	8635751	13	7924797	25	53,404
Lys	998817	6	971455	8	1029356	6	977326	13	963395	6	1078849	10	1104131	11	6784
Arg	410090	13	303775	19	454062	16	521586	19	358662	12	721408	16	785264	36	6771
hArg	28346	10	23085	19	31282	9	35135	20	26495	8	50562	16	51834	37	4700
Trp	209036	23	230573	18	267933	18	272679	19	241258	17	235670	20	209196	26	1058
ADMA	4694	15	4323	18	4632	36	5482	19	4408	8	6844	12	5519	38	3419

Table S2. Mean peak area (PA) values (in arbitrary units) of the amino acids, coefficient of variation (CV, %) values, in the study samples within eight runs as analyzed by GC-MS.

**AA**	Run 1		Run 2		Run 3		Run 4		Run 5		Run 6		Run 7		Run 8	
	PA	CV	PA	CV	PA	CV	PA	CV	PA	CV	PA	CV	PA	CV	PA	CV
Ala	274781	13	200142	30	200576	18	206892	9	150739	35	182721	9	138641	34	152276	23
Thr	396459	13	284616	32	295498	15	307398	9	218966	32	257921	10	200245	30	213076	21
Gly	691479	34	642019	51	493833	58	414219	48	331677	72	666503	29	388916	57	411854	45
Val	193093	12	157265	25	160828	15	162965	8	129391	28	142623	9	116464	28	130047	20
Ser	262789	47	278783	57	130607	90	99544	73	74354	96	153326	50	97535	66	103030	53
Sarc	41853	12	31216	32	34674	16	34491	11	24488	27	28537	11	22809	27	25047	19
Leu	429077	12	358659	23	344736	14	336521	8	288051	23	319598	6	273688	22	295686	15
GAA	264369	12	248034	20	231268	14	223475	7	219936	11	215902	5	201881	14	209943	4
Asp/Asn	322562	24	273380	32	211149	28	187261	36	134927	41	210094	16	147194	43	75602	81
OH-Pro	7168	30	7970	32	6000	46	5547	41	5613	43	10334	20	7495	37	7298	31
Pro	124063	11	109843	22	108236	14	103580	8	97189	12	103154	5	96463	15	95409	6
Glu/Gln	926495	17	874165	21	883686	15	858341	11	804031	13	854656	5	822360	15	866632	4
Met	311844	10	296702	13	310092	10	302883	6	294989	8	288799	4	286311	9	293830	3
Orn/Cit	1604174	12	1630165	22	1422623	14	1450100	6	1432099	17	1417299	4	1377350	17	1419037	5
Phe	256125	12	247016	17	232933	14	220250	9	220661	9	224074	6	215999	14	212843	6
Tyr	4373236	33	5269937	33	3910401	45	3460325	36	3349887	40	5943924	18	4604934	33	4686599	25
Lys	782749	13	803343	23	714907	15	721543	8	730777	19	732428	6	724943	18	736847	6
Arg	233291	25	209626	25	91264	46	48111	53	48666	49	86816	24	56021	52	59195	46
hArg	24923	24	23278	18	10858	38	5713	47	4658	56	8202	27	4541	81	4555	67
Trp	122249	38	193791	39	180927	35	151372	35	210025	35	379120	23	298910	34	251432	27
ADMA	2093	35	3160	39	2302	39	2256	40	2270	51	3653	21	2872	39	2636	25

Table S3. Median peak area (PA) values of the internal standards (IS) from the GC-MS analyses of the study samples (A) and the plasma quality control (B) samples, peak area ratio (PAR) range in the study samples (C) and QC samples (D), and mean of the PAR in (C) and (D). n.s., not significant.

AA	(A) Median PA of IS in the study samples	(B) Median PA of IS in the QC samples	Mann-Whitney *P* value	(A) / (B)	(C) PAR study samples [min - max]	(D) PAR QC samples [min-max]	(C) / (D) of mean PAR
Ala	191446	201644	0.0158	0.95	0.75 - 2.21	1.30 - 2.84	0.65
Thr	466170	926904	< 0.0001	0.50	1.59 - 5.12	3.18 - 4.48	1.36
Gly	274879	277537	0.2329	0.99	0.73 - 6.56	0.79 - 2.41	0.63
Val	151874	162566	< 0.0001	0.93	0.49 - 2.06	0.82 - 3.42	0.57
Ser	122530	264969	< 0.0001	0.46	0.28 - 1.59	0.48 - 2.04	0.39
Sarc	30519	34766	< 0.0001	0.88	0.66 - 3.36	0.20 - 1.49	2.39
Leu/Ile	327251	361001	< 0.0001	0.90	0.27 - 1.31	0.44 - 1.70	0.58
GAA	222252	257417	< 0.0001	0.86	0.04 - 0.43	0.07 - 0.27	0.34
Asp/Asn	195918	412639	< 0.0001	0.48	0.25 - 0.68	0.34 - 2.30	0.53
OH-Pro	7110	12974	< 0.0001	0.55	0.35 - 1.95	0.68 - 13.5	0.16
Pro	103098	120382	< 0.0001	0.86	2.37 - 12.9	4.65 - 19.4	0.49
Glu/Gln	861074	734609	< 0.0001	1.17	0.67 -1.77	1.44 - 10.2	0.33
Met	298236	191777	< 0.0001	1.55	0.66 - 1.34	0.99 - 2.94	0.56
Orn/Cit	1463942	1777970	< 0.0001	0.82	0.33 - 1.02	1.09 - 2.85	0.35
Phe	225072	266453	< 0.0001	0.85	0.32 - 1.09	0.52 - 1.94	0.45
Tyr	4414245	7935913	< 0.0001	0.56	0.27 - 1.17	0.61 - 1.77	0.57
Lys	743521	9993626	< 0.0001	0.74	0.87 - 3.94	1.13 - 2.88	0.83
Arg	75914	442521	< 0.0001	0.17	0.34 - 6.05	0.80 - 2.20	0.98 (n.s.)
hArg	8631	29357	< 0.0001	0.29	0.11 - 7.45	0.20 - 1.59	0.44
Trp	210320	228435	0.312	0.92	0.09 - 0.40	0.10 - 1.44	0.27
ADMA	2742	4857	< 0.0001	0.56	0.17 - 0.89	0.26 - 1.33	0.51
